# Direct current remote cloak for arbitrary objects

**DOI:** 10.1038/s41377-019-0141-2

**Published:** 2019-03-13

**Authors:** Tianhang Chen, Bin Zheng, Yihao Yang, Lian Shen, Zuojia Wang, Fei Gao, Erping Li, Yu Luo, Tie Jun Cui, Hongsheng Chen

**Affiliations:** 1 Key Laboratory of Micro-Nano Electronics and Smart Systems of Zhejiang Province, College of Information Science and Electronic Engineering, Zhejiang University, 310027 Hangzhou, China; 2 State Key Laboratory of Modern Optical Instrumentation and The Electromagnetics Academy at Zhejiang University, Zhejiang University, 310027 Hangzhou, China; 3 School of Electrical & Electronic Engineering, Nanyang Technological University, Singapore, 637371 Singapore; 4 State Key Laboratory of Millimeter Waves, Department of Radio Engineering, Southeast University, 210096 Nanjing, China

**Keywords:** Optics and photonics, Electronics, photonics and device physics

## Abstract

Hiding an arbitrary object with a cloak at a distance from an object is of great significance in scientific research, but remains unrealized as a practical device. In this paper, we propose the first experimental realization of a remote cloaking device that makes any object located at a certain distance invisible at direct current (DC) frequency. A negative resistor network with active elements is used to achieve the remote function of the DC cloak. Based on this network, the cloak can remotely generate a hidden region without distorting the currents far from the cloaked region. The experimental results show that any object in the hidden region is invisible to a DC detector. Our cloak does not require any knowledge of the hidden object. The experimental demonstration shows the superiority of this remote cloaking device, which may find potential applications in medical or geologic research.

The demand for making an object invisible to eyes or detectors inspires amazing cloak technology. With transformation optics^1^, a cloak could guide electromagnetic waves to bypass the cloaked region without any perturbation^2–11^. In previous works on cloaks^12–16^ and illusion devices^17–20^, the hidden object is usually enclosed by the cloaking device and hence cannot interact with the environment outside^21–23^. To solve this problem, a remote cloak that can hide an object from a distance was proposed based on the concept of an “anti-object”^24^. The scattering of the hidden object is exactly canceled by the “anti-object” at a distance while the hidden object keeps space continuity with the background environment.

However, this “anti-object” cloaking is designed for a hidden object with known parameters. Small changes in the shape, size, and position of the hidden object deteriorate the exact restoration of the incident field. Therefore, “anti-object” cloaking cannot hide arbitrary objects like the conventional internal cloak can. To avoid this limitation, a multi-folded transformation optics method was proposed to design remote cloaking that can hide objects of arbitrary shapes and materials^25^. However, such a design requires double-negative materials, which are very difficult to realize. In short, remotely hiding arbitrary objects is still at the conceptual stage and has not been experimentally demonstrated.

In this work, we propose the first experimental realization of a remote cloaking device that can hide an arbitrary object external to the cloak itself at direct current frequency. The remote direct current (DC) cloaking device is designed with multi-folded transformation optics. A negative resistor network with active elements is realized, which plays an important role in implementing the remote function of the DC cloak. The cloak, therefore, can remotely generate a hidden region without distorting the currents far from the cloaked region. The experimental results clearly show that different objects in the open hidden region are invisible.

Figure 1 shows the schematics of two kinds of cloaking at DC frequency. Figure 1a shows a closed cloak, while Fig. 1b shows remote cloaking at DC frequency. A remote cloak can be constructed with one element, as shown in Fig. 1b. A remote cloak can also be constructed with several elements instead. The parameters become more complicated if fewer elements are used to design the cloak. This remote cloak can be designed with multi-folded transformation optics^25^ at DC frequency.

**Fig. 1:**
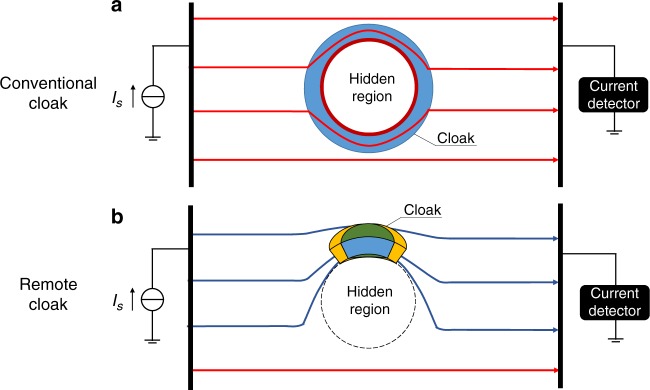
Schematics of two kinds of DC cloaking. **a** Conventional cloaking and **b** remote cloaking

In the following, a remote cloak with two elements is designed as an example. A detailed analysis of this methodology can be found in the Supplementary Information. The free space is first transformed into a square cloak^26^, and thereafter, a second transformation is applied to fold the square cloak to make it open. Then, the constitutive parameters for the DC cloak can be deduced. The hidden region, while still retaining space continuity with the background environment, is completely insulated from outside current fields. Therefore, any arbitrary static object in the hidden region or arbitrary object freely moving inside the hidden region can remain invisible. This phenomenon is completely different from previous DC cloaks based on resistor networks^27^ and DC exterior cloaks based on the ‘anti-object’’ theory^28^, where the performances of the cloaks depend on the shape and conductivity of the hidden object.

Figure 2 shows the simulation results of currents flowing from the point source through the cloak. The simulation is performed with the Finite Element Method analysis software COMSOL Multiphysics. We use a steady current-source at the top right corner of the simulation. The cloak is composed of two elements that are close to the hidden object. The background media has a conductivity of 15.783 S/m. Figure 2a, b show the simulated potential distribution for a uniform background and a circular insulator object, respectively. The presence of the object strongly affects the equal-potential contours. On the contrary, Fig. 2c shows that the remote cloak can effectively guide the current around the object, leaving the potential distribution undisturbed. For a clearer view, we extract the electric potential data from the position *y* = − *x* − 0.45(m) for the three cases to quantitatively demonstrate the cloaking effect. The results are plotted in Fig. 2d, which clearly shows that the potential distributions of the cloaking device and the background agree very well.

**Fig. 2:**
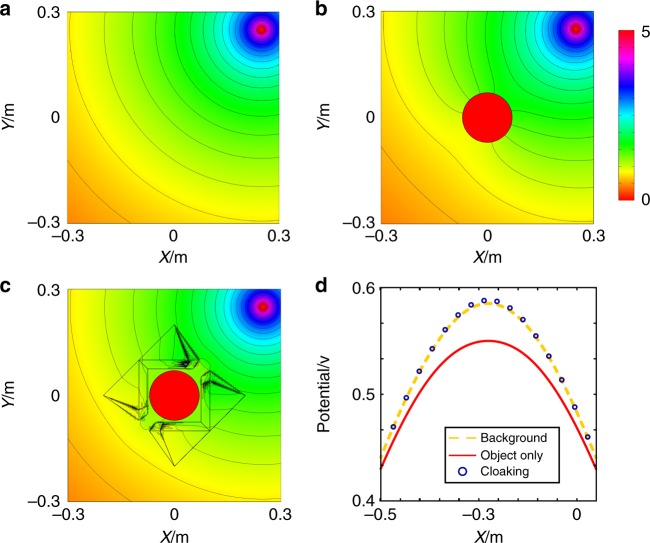
Simulated potential distribution with currents flowing from a point source in three different cases. **a** A homogeneous and isotropic background only, **b** a circular insulator as a hidden object, and **c** a hidden object with a remote cloak. **d** The electric potential on the line *y* = − *x* − 0.45(m). The purple circles and the orange dashed line represent the cloaking and background cases, respectively, while the red line is for the case of the object only

To verify the object-independent performance of the cloak, two additional different hidden objects, i.e., a square insulator (*σ* = 0) and a circular good conductor (with *σ* = 1×10^8^ S/m), are tested. The results are shown in Fig. 3a,  b, respectively. The measured electric potential is also compared with the cases of the background and the object only, respectively. The results are shown in the insets, which show that cases for the cloaking and the background are in excellent agreement, indicating that the cloaking performance is independent on the object.

**Fig. 3:**
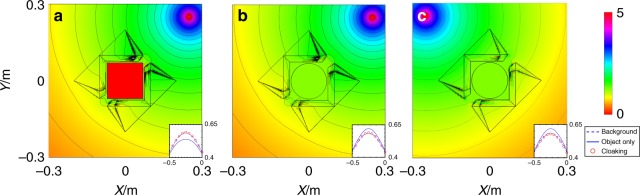
Simulated equipotential line patterns under different hidden objects or positions. The center object with **a** square insulator, **b** circular good conductor, and **c** when the source is placed at a different location

Furthermore, to demonstrate the omnidirectional effect of this remote cloak, we simulated the steady current-source in a different position with respect to the cloak. As shown in Fig. 3c, the cloaking device still works very well. Further results with various source distances can be found in the Supplementary Information. The simulations of Figs. 2 and 3 show that the two elements of the cloak do not have to be close to each other. When the distance between the cloak and the object increases, the cloak involves more negative parameters. As a result, the computational complexity and memory consumption are highly increased. The simulation shown here only gives an example to verify the concept of the remote cloak.

To experimentally demonstrate the above concept, the remote cloak sample is designed and fabricated. The cloak requires anisotropic and negative conductivity to realize the complementary media in certain regions (see Supplementary Information). The required anisotropic conductivity is designed with ‘mesh-based’’ transformation optics^29,30^, while the negative conductivity is designed with a negative medium module composed of active elements. In the following, we show how this equivalent module functions.

DC negative conductivity material can provide potential ‘rise’’ when current traverses the material, which means that in Fig. 4a, the two-port network satisfies *V*_a_ > *V*_b_. When the current *I* is known, the two-port network can be reproduced by the impedance match shown in Fig. 4b. Analyzed from port A, the potential ‘rise’’ can be equivalently realized by source *V*_s_ and matching impedance $R_{\mathrm{a}} = \frac{{V_{\mathrm{s}} - V_{\mathrm{a}}}}{I}$, while from port B the equivalent circuit is composed of matching impedance $R_{\mathrm{b}} = \frac{{V_{\mathrm{b}}}}{I}$ and a grounded ‘source’’ as every parameter is determined^31^. Further, at DC frequency, the combination of resistor and source can be simplified to a single source with a power supply, as shown in Fig. 4c. To realize such effective negative media, we provide the required electric potential with a voltage follower. With a pair of voltage followers, the subsystem is able to provide the potential required for the negative resistor. The whole negative region is divided into 124 legs of bleeder circuit. A detailed design and photo of the fabricated Printed Circuit Board are shown in the Supplementary Information. Two additional rheostats are added to each leg for a possible minor tune of the bleeder circuit from any interference.

**Fig. 4:**
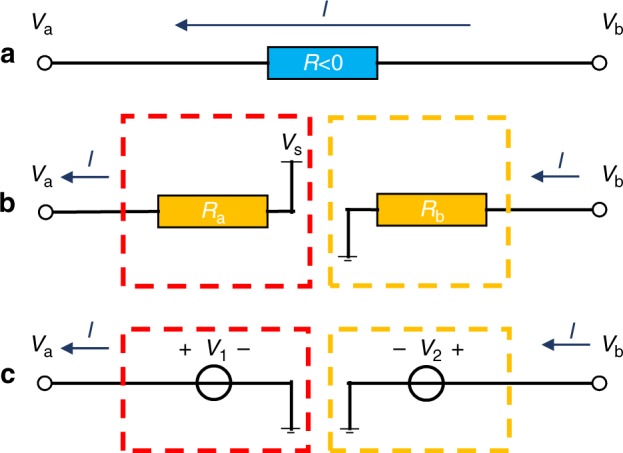
Schematic of the effective negative media (resistor). **a** Ideal negative resistor. **b** Equivalent negative resistor by applying the impedance match module. **c** Simplified two-source module

To verify the performance of our design, the whole circuit board of the cloak is fabricated with a size of 60  × 60 cm. To achieve a sharper potential drop for observation, we set the boundary as a circular conductor plate with a radius of 1.2 m. Boundary matching resistors are calculated approximately by $R = \frac{{\varphi _{{\mathrm{boundary}}}}}{{i_{{\mathrm{out}}}}}$, where *i*_out_ can also be calculated with numerical arithmetic^27^. The circuit is designed the same as that in the above discussion of an equivalent DC circuit that is based on the 330 Ω resistor network background.

We achieve the required electric conductivity with surface mounted device (SMD) resistors. The SMD resistors can be pasted by surface mounted technology that is able to fabricate complicate circuits with full automation. The experimental device is shown in Fig. 5a. The negative media, cloaked object, and boundary matching are designed with independent circuit boards separated from the main board for easy replacement by other circuits. As shown in Fig. 5b–d center, the hidden object can be replaced with other imitated object circuits in the real experiment.

**Fig. 5:**
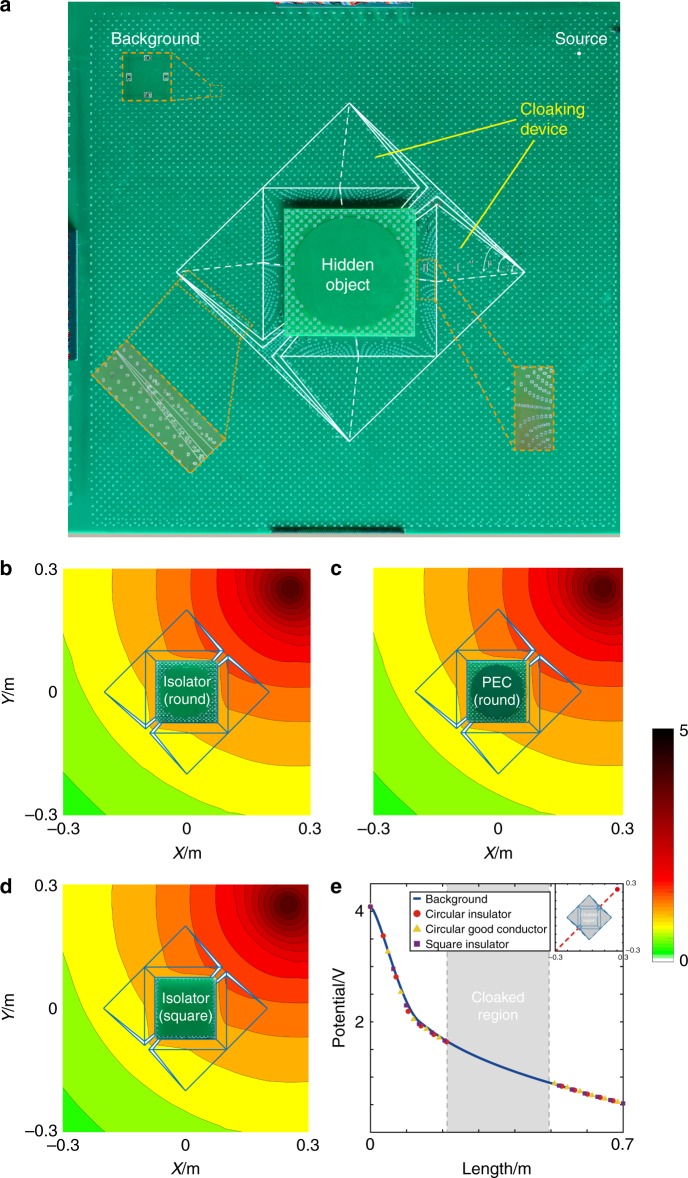
**a** Experiment set-up. **b**–**d** The measured potential distributions in experiments when currents flow from a point source through the remote cloak with three different objects: **b** circular insulator, **c** circular good conductor (PEC), and **d** square insulator. **e** The measured electric potential on the line *y* = *x*(m) compared with a no-object background. The circle, triangle, and square dashed lines are for the three cloaking cases, respectively

The measured results for three different hidden objects, a circular insulator, circular good conductor, and square insulator, are shown in Fig. 5b, c, d, respectively. These results show that the equipotential lines are very ‘round’’ as if nothing is there, which shows our design has worked properly to cancel the distortion caused by different hidden objects, indicating good cloaking functionality. For qualification, we analyzed the electric potential decay from the source in the direction *y* = *x*(m) for three experiments. As shown in Fig. 5e, the results agree well with the no-object background.

In the experiment, we use active circuits to mimic the negative resistor, as shown in Fig. 4. In these active circuits, we monitor the current flowing through the active circuits and then apply an inverse voltage to realize the negative resistance. This manual monitoring system is, therefore, dependent on the source. However, if the active system can automatically monitor the current and output an inverse voltage correspondingly in the fabricated device, the negative resistor will be ideal. The cloak will be independent of the source. Although the realization of the negative resistance in our experiment is not ideal based on current state of the art technology, the measured results in Fig. 5 prove that the performance of the proposed remote cloak is independent of the object.

In conclusion, we experimentally demonstrate for the first time a remote cloak that works for arbitrary objects at a distance at DC frequency. The cloak is designed with multi-folded transformation optics and is independent of the object. As all the electronic components we use are DC static elements, the cloak is much more stable than those with high frequencies. With the help of active elements, such a cloak can guide electric currents around a hidden object, while the object maintains a physical connection with the outside space. This feature provides certain potential application scenarios: arbitrary objects could be buried underground with a deployed cloaking device at a distance and the object could be invisible under geologic current sensors. Medical personnel may also apply such a method to avoid interference to instruments in vivo remotely from current probes. Furthermore, this method may also warn medical personnel that active elements around pathological organizations could cause misdiagnosis in ACEIT (Applied Current Electrical Impedance Tomography) technologies. Thus, we expect the proposed cloaking device to provide guiding significance to and find potential applications in medical and geologic research.

## Methods

### Transformation optics on DC

We start from the following continuity equation of electric current:

1 $$\frac{{\partial \rho }}{{\partial t}} + \nabla \cdot \overline J = F(x,y)$$

where *F*(*x,y*) is the current-source density. For a steady field with $\frac{{\partial \rho }}{{\partial t}} = 0$, the continuity equation for steady electric current becomes:

2 $$\nabla \cdot \overline J = F(x,y)$$

For the scalar potential *φ* with $\overline E = - \nabla \varphi$ and the Ohm law $\overline j = \sigma \overline E$, we can derive the equation of the direct current field:

3 $$\nabla ^2\varphi = - f(x,y)$$

where $f(x,y) = F(x,y)/\sigma$ Our detailed transformation can be found in the Supplementary Information.

### Mesh-based transformation optics

Unlike previous works using cylindrical coordinate transformation to build the basic mesh, our cloaking device is designed based on transformation in Cartesian coordinates. Additionally, the multi-folded transformation applied in the cloak needs a finer mesh with more resistors per unit area. The transformation results in a very complex resistor distribution that is extremely difficult to implement. To further reduce the number of types of resistors used in the experiment, “mesh-based” transformation optics are used to create nonorthogonal grids of resistors. The grids are a direct result of the transformation functions that require only uniform resistors.

The detailed derivations can be found in the Supplementary Information.

## Supplementary information

Supporting Information.docx
