# Supplementary material for: Direct current remote cloak for arbitrary objects
Source: Light Sci Appl. 2019 Mar 13;8:30. doi: 10.1038/s41377-019-0141-2 (PMC6414663; doi:10.1038/s41377-019-0141-2)
Supplement: Supplementary file 1 — Supporting Information.docx [file 41377_2019_141_MOESM1_ESM.docx]

Supplementary Information

Direct current remote cloak for arbitrary objects

Tianhang Chen^1, 2^, Bin Zheng^1, 2, *^, Yihao Yang^1, 2, *^, Lian Shen^1, 2^, Zuojia Wang^1, 2^, Fei Gao^1, 2^, Erping Li^1, 2^, Yu Luo^3^, Tie Jun Cui^4^, Hongsheng Chen^1, 2, *^

^1^Key Laboratory of Micro-Nano Electronics and Smart Systems of Zhejiang Province, College of Information Science and Electronic Engineering, Zhejiang University, Hangzhou 310027, China

^2^State Key Laboratory of Modern Optical Instrumentation and The Electromagnetics Academy at Zhejiang University, Zhejiang University, Hangzhou 310027, China

^3^School of Electrical & Electronic Engineering, Nanyang Technological University, Singapore 637371, Singapore

^4^State Key Laboratory of Millimeter Waves, Department of Radio Engineering, Southeast University, Nanjing 210096, China

Official email addresses of all the authors:

Tianhang Chen (greendam@zju.edu.cn)

Bin Zheng (zhengbin@zju.edu.cn)

Yihao Yang (yangyihaooo@zju.edu.cn)

Lian Shen (shenlian918@hotmail.com)

Zuojia Wang (jacky012@zju.edu.cn)

Fei Gao (gaof@ntu.edu.sg)

Erping Li (liep@zju.edu.cn)

Yu Luo (luoyu@ntu.edu.sg)

Tie Jun Cui (tjcui@seu.edu.cn)

Hongsheng Chen (hansomchen@zju.edu.cn)

Full contact details of the corresponding authors:

Bin Zheng (zhengbin@zju.edu.cn) Telephone: 86-0571-87951013

Yihao Yang (yangyihaooo@zju.edu.cn) Telephone: 86-0571-87951013

Hongsheng Chen (hansomchen@zju.edu.cn) Telephone: 86-0571-87951013

**Transformation optics on DC**

We start from the following continuity equation of electric current:

$\frac{\partial\rho}{\partial t}+\nabla\cdot\overline{J}=F(x,y)$ (S1)

where $F(x,y)$is the current-source density. For a steady field with $\frac{\partial\rho}{\partial t}=0$, the continuity equation for steady electric current becomes:

$\nabla\cdot\overline{J}=F(x,y)$ (S2)

For the scalar potential $\varphi$with and the Ohm law$\overline{J}=\sigma\overline{E}$,we can derive the equation of direct current field:

(S3)

where $f(x,y)=F(x,y)/\sigma$.

According to the above analysis, we use a steady current source to excite the field and measure the potential to demonstrate the DC cloak.

**The schematic of transformation**

The transformation schematics of the designed DC cloak are shown in Fig.S1. Similar to the theory of remote cloaking that can hide arbitrary object proposed for electromagnetic wave^1^, we started our transformation based on the conventional square cloak^2^ to fold the quadrilateral area to its narrower edges. Hereafter, according to the multi-folded transformation optics theory, the second transformation is applied to keep the square cloak at a distance from the cloaked object. Fig. S1(a) and S1(b) show the meshes before and after the transformation, respectively. For detail, as shown in Fig. S1(c), we compress the $\left(-\frac{\pi}{4}\text{，}\frac{\pi}{4}\right)$ region towards the line $y=x-2a$and fold the negative media backward to complement the background area generated by the compression. For a uniform background media with the conductivity of $1S/m$, the transformation equations and the conductivity tensors for each region in the$\left(-\frac{\pi}{4}\text{，}\frac{\pi}{4}\right)$segment are as follows:

For region I, the transformation functions are:

$\begin{matrix} x'=x \\ y'=\kappa\left[y-(x-2a)\right]+(x-2a) \end{matrix}$ (S4)

where $\text{κ}$ is the compression ratio and $\kappa=\frac{1+\tan\gamma}{2}$. The conductivity tensor for region I can then be obtained:

$\sigma_{I}=\left(\begin{matrix} \frac{1}{\kappa} & \frac{1-\kappa}{\kappa} \\ \frac{1-\kappa}{\kappa} & \frac{(\kappa-1)^{2}+\kappa^{2}}{\kappa} \end{matrix}\right)$ (S5)

Based on the square cloak^2^, the transformation functions for region II are

$\begin{matrix} x'=x\left(\frac{r_{2}-r_{1}}{r_{2}}\right)+r{}_{1} \\ y'=\left[\kappa(y+x)-x\right]+(\frac{r_{2}-r_{1}}{r_{2}}+\frac{r_{1}}{x}) \end{matrix}$ (S6)

where $r_{1}$ is half sidelength of the inner square,$r_{2}$ is the half sidelength of the outer square.The conductivity tensor for region II is:

$\sigma_{II}=\left(\begin{matrix} \frac{-(r_{1}-x)}{\kappa x} & -\frac{r_{1}y+(1-\kappa)x^{2}}{\kappa x^{2}} \\ -\frac{r_{1}y+(1-\kappa)x^{2}}{\kappa x^{2}} & \frac{2\kappa x^{4}(\kappa-1)-2r_{1}x^{2}y(\kappa-1)+r_{1}^{2}y^{2}+x^{4}}{\kappa x^{3}(r_{1}-x)} \end{matrix}\right)$ (S7)

For regions III and IV, the transformation functions are:

$\begin{matrix} x'=x \\ y'=-y+2\tau\left(P-x\right) \end{matrix}$(for region III) (S8)

and

$\begin{matrix} x'=x \\ y'=-y+2\tau\left(x-P\right) \end{matrix}$ (for region IV) (S9)

Where,$\tau=\tan\beta=\frac{\tan\alpha+\tan\gamma}{2}$ is the slope for the folding transformation and $P=2a$is as the intersection point as Fig.S1(c).

The conductivity tensors for region III and IV are:

$\sigma_{III}=\left(\begin{matrix} -1 & 2\tau\\ 2\tau& -(4\tau^{2}+1) \end{matrix}\right)$(for region III) (S10)

and

$\sigma_{IV}=\left(\begin{matrix} -1 & -2\tau\\ -2\tau& -(4\tau^{2}+1) \end{matrix}\right)$ (For region IV) (S11)

In addition, due to the symmetry of the cloak, the conductivity tensors for other segments as shown in Fig. S1(c) can be obtained by rotating the parameters above by$\frac{\pi}{2}$, $\pi$and$\frac{3\pi}{2}$, respectively.

In experiments, both $\kappa$ and $\text{τ}$ are set to be 0.7, with$\tan\alpha$, $\tan\beta$ and $\tan\gamma$ to be 1, 0.7, 0.4, respectively. It should also be noted that theoretically, $\kappa$ can be set smaller to make the gap of cloak wider.


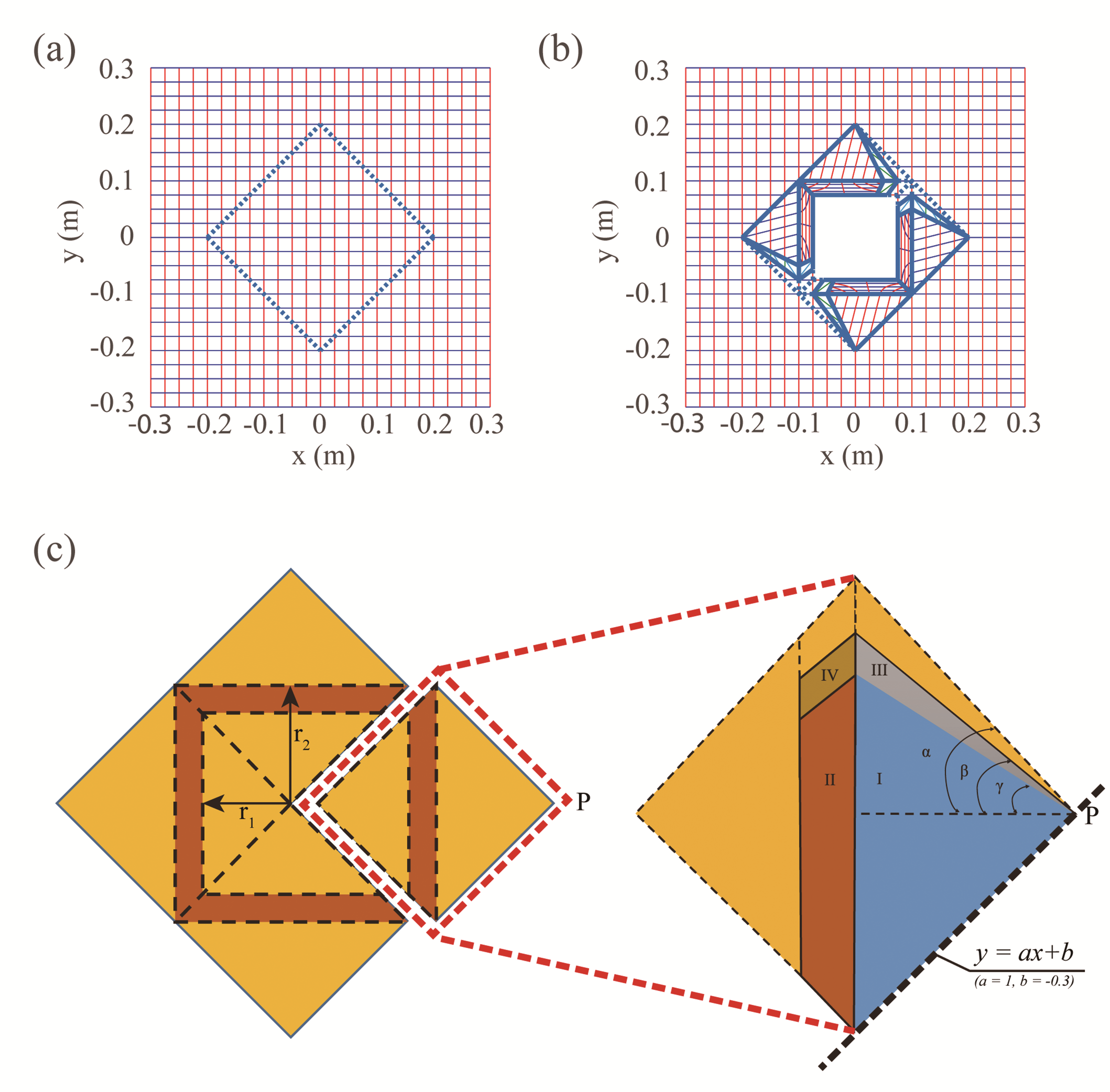


**Figure S1.** The schematic of the transformation applied. (a) Meshes for the virtual space with background media. (b) Meshes for the physical space after defined multi-folded transformation. (c) The schematic of the transformation from the conventional square cloak to the remote cloak at the segment of $\left(-\frac{\pi}{4}\text{，}\frac{\pi}{4}\right)$.

**Mesh-based transformation optics**

Unlike previous works^3–5^ using cylindrical coordinate transformation to build the basic mesh, our cloaking device is designed based on transformation on the Cartesian coordinate. Besides, the multi-folded transformation applied in the cloak needs finer mesh with more resistors per unit area. It results in very complex resistors distribution which is extremely difficult for implementation.

To reduce the complexity, the matrix diagonalization can be applied. Taking $\sigma_{1}$ for an example, we can rotate the local coordinate system by θ to fulfill the diagonal conductively (eq.S5). For example, in experiments, we set $\kappa=0.7\text{, }a=1$, $\sigma_{I}=\left(\begin{matrix} 1.4286 & 0.4286 \\ 0.4286 & 0.8286 \end{matrix}\right)$, then we can inversely calculate the rotation angle$\theta=27.504^{\circ}$andthe conductor tensor $\sigma_{I}'=\left(\begin{matrix} 1.652 & 0 \\ 0 & 0.605 \end{matrix}\right)$, by using$\sigma_{1}'=R^{T}\sigma_{1}R$ and $R=\left(\begin{matrix} \cos\theta& -\sin\theta\\ \sin\theta& \cos\theta\end{matrix}\right)$.

From the derivation above, one can see that in order to fulfill the required anisotropic electric conductivity, there should be two types of resistors arranged with a rotation angle relative to the original coordinate system. As shown in Fig. S2(b), the grid after transformation is still an orthogonal grid but with non-uniform resistors.Since there are four different regions derived from different transformations, it makes such conventional transformation meshes extremely difficult in implementation.

In order to further reduce the numbers of types of resistors used in the experiment, the “mesh-based” transformation optics are used to create non-orthogonal grids of resistors. It is a direct result from the transformation functions that requires only the uniform resistors. Still taking $\sigma_{1}$ for example, for the transformation$y'=\kappa\left[y-(ax-b)\right]+(ax-b)$, the transformed resistors will only change the location instead of varying its value. As the same condition above when $\kappa=0.7\text{, }a=1$, the transformation becomes$y'=0.3(x-b)+0.7y$. The grid after transformation is shown in Fig. S2(c), from which one can see that the resistors along the x-axis after transformation will have a slight rotation angle of while resistors along the y-axis remain unchanged. Furthermore, it requires only uniform resistors arrays in such transformation meshes which can reduce the complexity in the experimental realization.


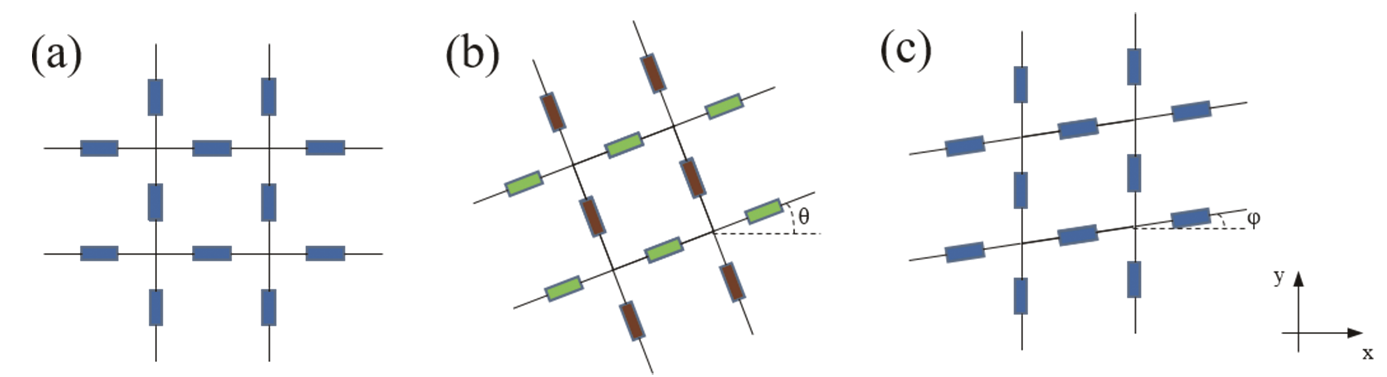


**Figure S2.** The schematic of the two transformation methods. (a) The original resistor network mesh. (b) Conventional orthogonal transformation mesh. (c) The mesh-based transformation network mesh(non-orthogonal).

**Different source distance**

Additional simulations of different source distance are performed. We provide the simulation results when the source is moved to $(-0.29,0.29)$and $(-0.2,0.2)$ in Fig.S3 (a) and (b), respectively. The value of $\frac{V_{cloak}}{V_{background(ideal)}}$at the position of $y=x-0.45$(m) are illustrated in Fig.S3 (c) which indicates the cloak still working at an ideal status as source varies.


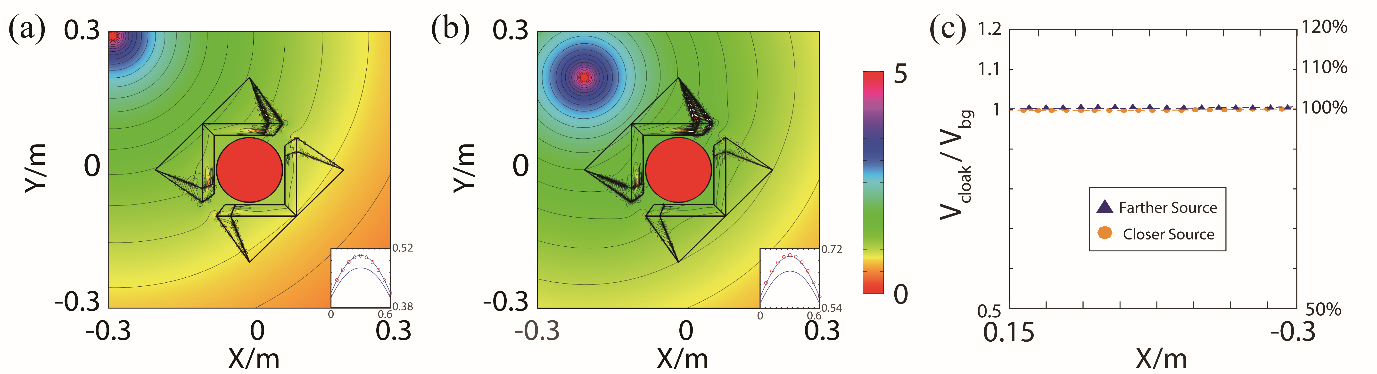


**Figure S3.** Simulated equipotential line pattern under (a)a farther source and (b)a closer source. (c) The value of $\frac{V_{cloak}}{V_{background(ideal)}}$ under the circumstances of (a) and (b).

**Voltage follower array**

A voltage follower can be seen as an op-amp circuit which has a voltage gain of 1. With high input impedance and low output impedance, a voltage follower can ‘copy’ the electric potential from a simple bleeder circuit to easily achieve any acquired voltage. The schematic of the design is shown in Fig.S4(a). Fig. S4(b) shows the fabricated PCB sub circuit. In the experiment, four circuit boards are applied to fulfill all the negative resistors.


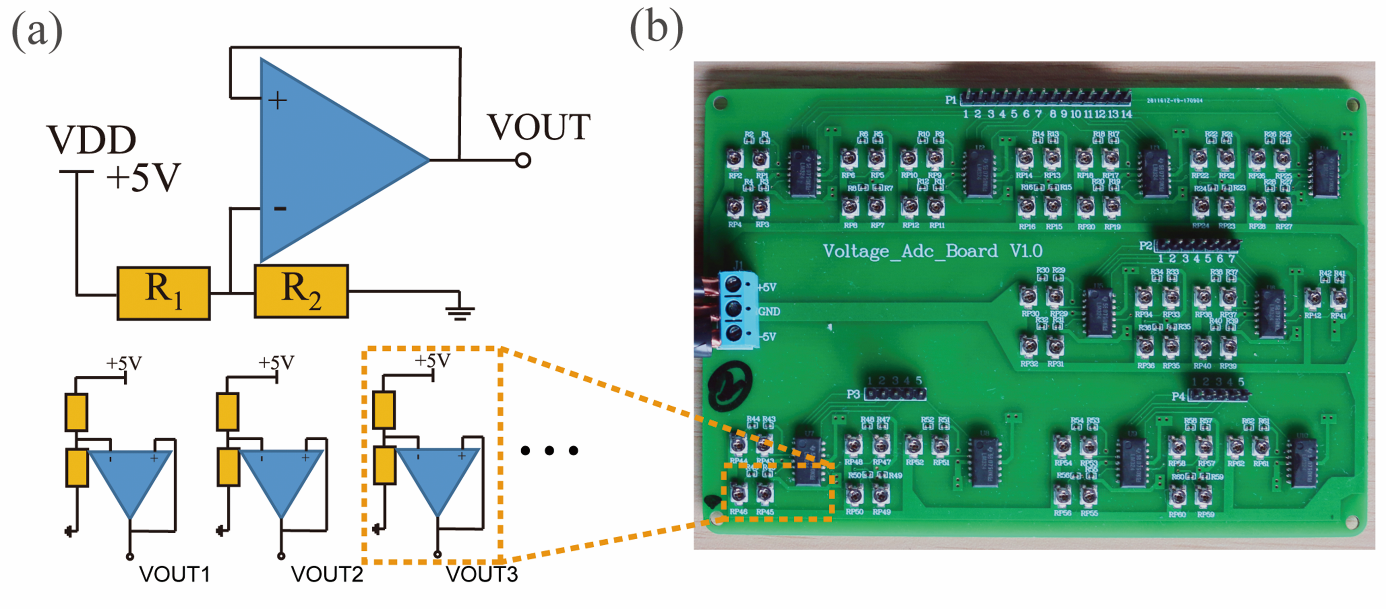


**Figure S4.** The circuit design of negative media at direct current frequency. (a) The schematic of the voltage-follower based bleeder circuit array. (b) The fabricated PCB sub circuit with 31 legs of equivalent negative resistors.

**References**

1. Zheng, B. *et al.* Concealing arbitrary objects remotely with multi-folded transformation optics. *Light Sci. Appl.***5,** e16177 (2016).

2. Rahm, M. *et al.* Design of Electromagnetic Cloaks and Concentrators Using Form-Invariant Coordinate Transformations of Maxwell’s Equations. *Photonics Nanostructures - Fundam. Appl.***6,** 87–95 (2007).

3. Yang, F., Mei, Z. L., Jin, T. Y. & Cui, T. J. dc Electric Invisibility Cloak. *Phys. Rev. Lett.***109,** 053902 (2012).

4. Yang, F., Mei, Z. L., Yang, X. Y., Jin, T. Y. & Cui, T. J. A negative conductivity material makes a dc invisibility cloak hide an object at a distance. *Adv. Funct. Mater.***23,** 4306–4310 (2013).

5. Ma, Q., Mei, Z. L., Zhu, S. K., Jin, T. Y. & Cui, T. J. Experiments on Active Cloaking and Illusion for Laplace Equation. *Phys. Rev. Lett.***111,** 173901 (2013).
